# Effect of hypoxia-inducible factor-1 alpha expression on survival in patients with metastatic cervical squamous cell carcinoma treated with first-line chemotherapy and bevacizumab

**DOI:** 10.17305/bb.2024.10255

**Published:** 2024-08-01

**Authors:** Hasan Cagri Yildirim, Hicran Anik, Deniz Ates Ozdemir, Rashad Ismayilov, Arif Akyildiz, Kerim Cayiroz, Fahri Ceyhan, Oguzalp Kavruk, Deniz Can Guven, Ozturk Ates, Alp Usubutun, Zafer Arik

**Affiliations:** 1 Department of Medical Oncology, Hacettepe University Cancer Institute, Ankara, Turkey; 2 Department of Medical Oncology, Dr. Abdurrahman Yurtaslan Training and Research Hospital, Ankara, Turkey; 3 Department of Pathology, Hacettepe University Faculty of Medicine, Ankara, Turkey; 4 Department of Internal Medicine, Hacettepe University Faculty of Medicine, Ankara, Turkey

**Keywords:** Metastatic cervical cancer, squamous cell carcinoma (SCC), vascular endothelial growth factor (VEGF), hypoxia-inducible factor-1 alpha (HIF-1 alpha), bevacizumab

## Abstract

This study addresses the gap in understanding the prognostic relevance of hypoxia-inducible factor-1 alpha (HIF-1 alpha) expression in metastatic cervical squamous cell carcinoma (SCC) patients undergoing anti-vascular endothelial growth factor (VEGF)-based therapy. A retrospective multicenter study (*n* ═ 34) explored HIF-1 alpha expression via immunohistochemistry in patients treated with platinum chemotherapy and bevacizumab. Median progression-free survival (PFS) was significantly lower in the HIF-1 alpha low score group compared to the high score group (4.9 vs 12.9 months, *P* ═ 0.014). Similarly, the median overall survival (OS) was significantly reduced in the HIF-1 alpha low score group (8.3 vs 20.4 months, *P* ═ 0.006). This study, the first of its kind, highlights the prognostic significance of HIF-1 alpha expression in metastatic cervical SCC patients treated with bevacizumab-based therapy.

## Introduction

Cervical cancer is the fourth most prevalent cancer among women but is preventable through primary or secondary prevention strategies [1]. Eighty-five percent of cervical cancer cases occur in developing countries, representing a major cause of mortality in these areas [2, 3]. Persistent HPV infection is responsible for more than 99% of cervical cancers, with 75% attributed to high-risk HPV types 16 and 18 [4]. HPV infection primarily activates E6 and E7 oncoproteins, subsequently triggering the PI3K/AKT/mTOR pathway through *p53* degradation and inducing the retinoblastoma tumor suppressor gene [5, 6]. In hypoxic conditions, E7 oncoprotein increases hypoxia-inducible factor-1 alpha (HIF-1 alpha) expression via ROS, ERK1/2, and NF-κB [4, 7]. The E6 oncoprotein contributes to tumor biology by preventing the degradation of HIF-1 alpha [8]. HIF-1 alpha influences many cancer hallmarks, including cellular proliferation, apoptosis, metabolism, immunological responses, genomic instability, vascularization, neovascularization, invasion, and metastasis [9]. This transcription factor is thought to play a role in the expression of many genes, including the vascular endothelial growth factor (VEGF), in hypoxic conditions [10]. VEGF family proteins and receptors also play a crucial role in tumor angiogenesis, and bevacizumab, an anti-VEGF monoclonal antibody, is used in the treatment of cervical squamous cell carcinoma (SCC) [11]. The integration of bevacizumab with chemotherapy has established itself as the standard approach for treating metastatic cervical SCC. This is due to evidence showing that adding bevacizumab to conventional chemotherapy regimens enhances overall survival (OS) in these patients [12]. However, complications, such as hypertension, thromboembolic events, or gastrointestinal fistula increase with the addition of bevacizumab [13]. In the Keynote 826 study, which subsequently set the new standard, more than 35% of the patients did not use bevacizumab, and no relationship was found between bevacizumab receipt and progression-free survival (PFS) [14].

While studies have explored the connection between the effectiveness of concurrent chemotherapy and radiotherapy and HIF-1 alpha expression in locally advanced cervical cancer, research on how HIF-1 alpha expression influences the prognosis of metastatic cervical cancer patients undergoing anti-VEGF-based therapy is absent [15]. In our study, we aimed to explore the impact of HIF-1 alpha expression levels on the survival of patients diagnosed with metastatic cervical SCC who were receiving chemotherapy in combination with bevacizumab as their initial treatment.

## Materials and methods

### Selection of patients

This retrospective multicenter study comprises patients from two distinct tertiary oncology hospitals, who were admitted between March 2015 and June 2023. Inclusion criteria were: being over 18 years old, having a diagnosis of metastatic cervical SCC, receiving a first-line treatment combining a platinum-based chemotherapy doublet with bevacizumab, and having formalin-fixed paraffin-embedded blocks available for analysis. Demographic characteristics and Eastern Cooperative Oncology Group-performance status (ECOG-PS) of the patients, type of metastatic disease (de novo or recurrence), metastasis sites, previous treatments, progression, and death data were recorded.

### Immunohistochemical procedure and evaluation

Sections 5µ thick were prepared from the paraffin-embedded tissue samples collected prior to initiating treatment with bevacizumab combined with chemotherapy. After deparaffinization, appropriate positive and negative controls were prepared, and HIF-1 alpha (Clone: EP118, Rabbit monoclonal antibody, Ready-to-use, Bio SB, Santa Barbara, CA, USA) primary antibody was used. Leica Bond Max (Shandon, Frankfurt, Germany) autostainer was employed for immunohistochemical procedures. For the evaluation of HIF-1 alpha, the well-known and long-established H scoring was used in immunohistochemistry scoring [16, 17]. Two senior pathologists collaborated on the evaluation. The H-score was a method of assessing the extent of nuclear immunoreactivity. Accordingly, the intensity scores ranged from 1–2 (low) to 3 (high), and the percentage of stained cells was mathematically multiplied. The score was obtained by the formula: 3 × percentage of strongly staining nuclei + 2 × percentage of moderately staining nuclei + percentage of weakly staining nuclei. These results were shown in a numerical range of 1–300.

### Ethical statement

Ethical approval for this study was obtained from the Hacettepe University Clinical Research Ethics Board (Decision no: 2022/20-07, Date: 29.11.2022). All procedures and stages of the study were conducted in compliance with the ethical principles outlined in the World Medical Association Declaration of Helsinki, which governs the inclusion of human subjects in medical research.

### Statistical analysis

Descriptive statistics were presented as frequency (percent), mean ± standard deviation (SD), or median (min–max). OS was defined as the time from the start of first-line metastatic disease treatment until death from any cause, and PFS was defined as the time until death or progression. Survival estimates were calculated with the Kaplan–Meier method. The log-rank test was used to identify the independent effects on PFS and OS. Possible risk factors for survival were investigated by univariate Cox regression analyses. A value of *P* < 0.05 was considered statistically significant.

## Results

### Characteristics of patients

The study included 34 women with a mean age of 55.1 ± 11.6 years (range 31–79). ECOG-PS was 0 in 4 (11.8%) patients, 1 in 18 (52.9%) patients, and 2 in 12 (35.3%) patients. Ten (29.4%) patients had de novo, while 24 (70.6%) patients had recurrent metastatic disease. Metastasis sites were soft tissue (50%), lung (32.4%), liver (26.5%), bone (23.5%), peritoneum (17.6%), brain (2.9%), colon (2.9%), and skin (2.9%), respectively. Twelve (35.3%) patients underwent surgery, and 23 (67.6%) patients received radiotherapy (Table 1).

**Table 1:** Characteristics of patients

**Characteristics**		**Frequency, *n* = 34 (%)**
Age (years), mean ± SD	55.1 ± 11.6
≥ 65 years		5 (14.7)
ECOG-PS	0	4 (11.8)
	1	18 (52.9)
	2	12 (35.3)
Type of metastatic disease	De novo	10 (29.4)
	Recurrence	24 (70.6)
Site of metastasis	Soft tissue	17 (50)
	Lung	11 (32.4)
	Liver	9 (26.5)
	Bone	8 (23.5)
	Peritoneum	6 (17.6)
	Brain	1 (2.9)
	Colon	1 (2.9)
	Skin	1 (2.9)
Surgical treatment		12 (35.3)
Radiotherapy		23 (67.6)

ECOG-PS: Eastern Cooperative Oncology Group-performance status; SD: Standard deviation.

### HIF-1 alpha score

**Figure 1.**
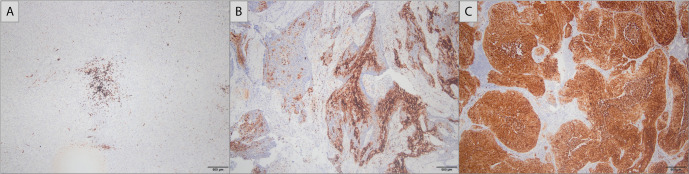
**Immunohistochemistry.** (A) HIF-1 alpha score 30 (40×); (B) HIF-1 alpha score 120 (40×); (C) HIF-1 alpha score 300 (40×). HIF-1: Hypoxia-inducible factor-1.

The median HIF-1 alpha score was 60 (0–300). It was 0 in 4 (11.8%) patients, between 1–99 in 16 (47.1%) patients, between 100 and 199 in 8 (23.5%) patients, and ≥ 200 in 6 (17.6%) patients (Figure 1A–1C).

**Figure 2.**
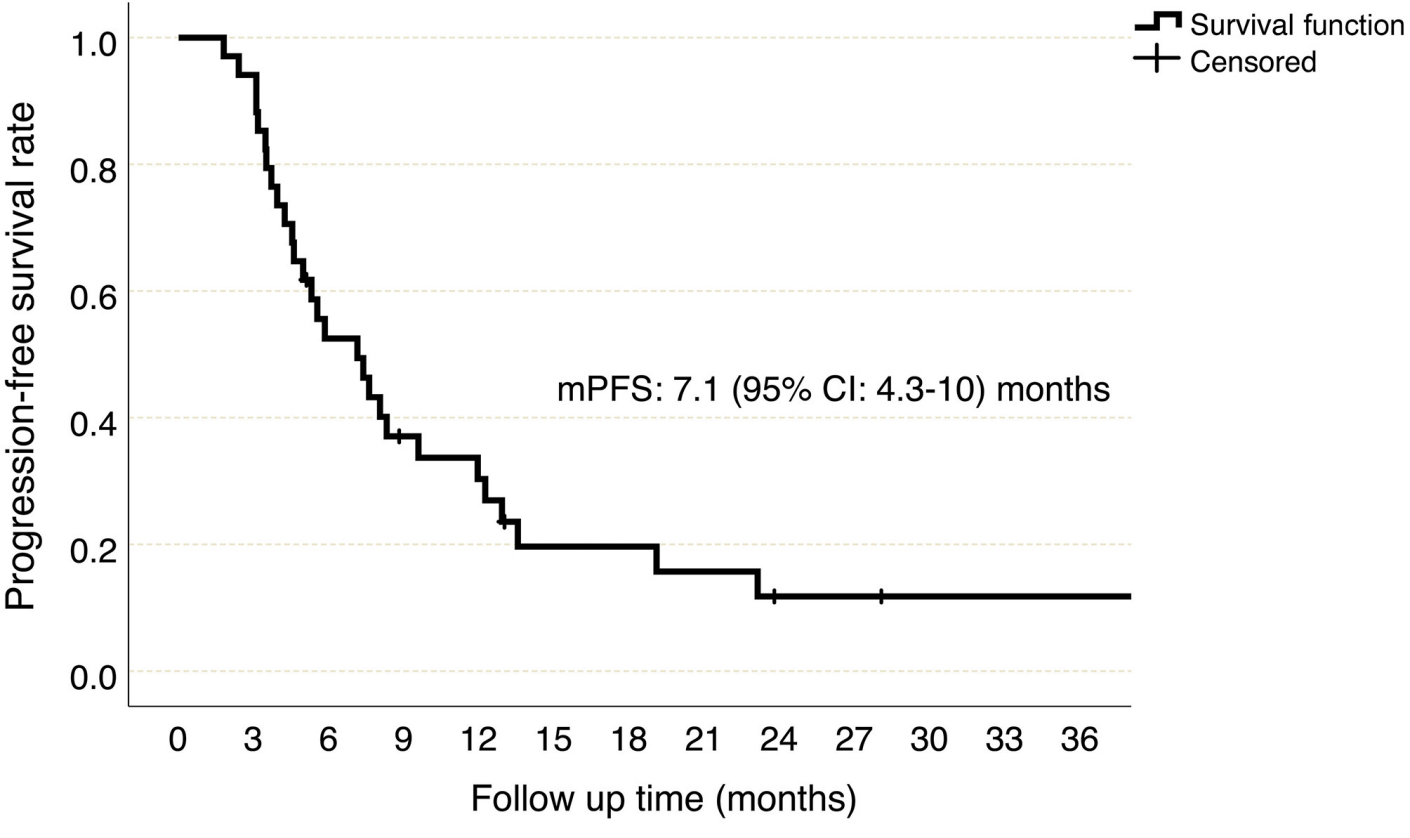
**Progression-free survival, Kaplan–Meier curve**. CI: Confidence interval; mPFS: Median progression-free survival.

### Survival outcomes

**Figure 3.**
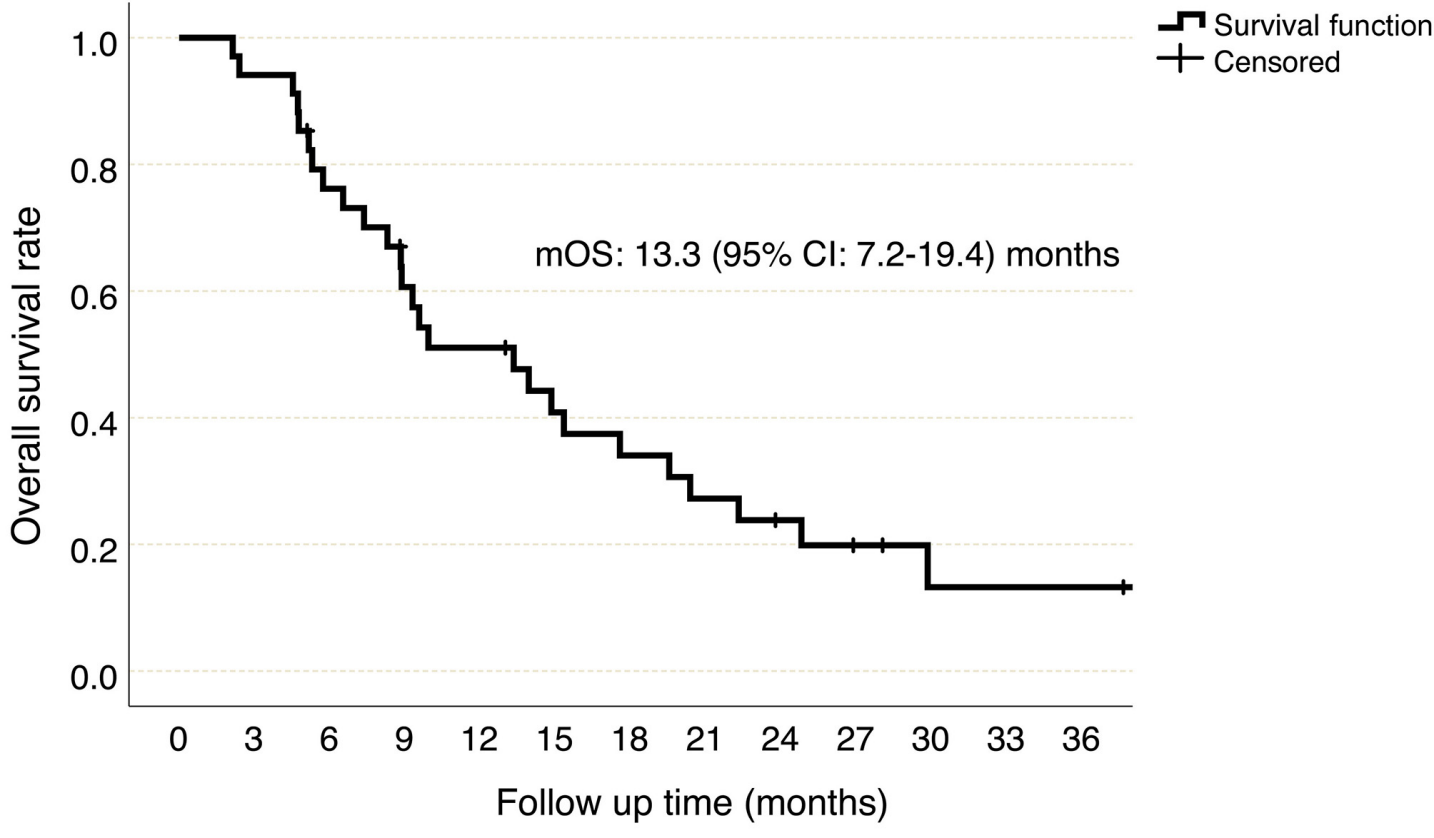
**Overall survival, Kaplan–Meier curve**. CI: Confidence interval; mOS: Median overall survival.

During the median follow-up period of 9.7 (range 2.1-38.6) months, 28 (82.4%) patients experienced progression and 26 (76.5%) of them died. The median PFS and OS were 7.1 (95% CI 4.3–10) and 13.3 (95% CI 7.2–19.4) months, respectively (Figures 2 and 3). In univariate analyses, age ≥ 65 years (*P* ═ 0.493), ECOG-PS 2 (compared to 0–1, *P* ═ 0.215), recurrent metastatic disease (compared to de novo metastatic disease, *P ═* 0.616), presence of soft tissue (*P* ═ 0.200), lung (*P* ═ 0.073), liver (*P* ═ 0.244), bone (*P* ═ 0.592) or peritoneal metastases (*P* ═ 0.625), undergoing surgery (*P* ═ 0.446), or receiving radiotherapy (*P* ═ 0.460) were not associated with an increased risk of progression. The OS was compatible with PFS (Table 2).

**Table 2:** Risk factors for PFS and OS

**Risk factors**		**PFS**			**OS**		
		**mPFS (95% CI), months**	**HR (95% CI)**	*P* value*	**mOS (95% CI), months**	**HR (95% CI)**	*P* value*
Age	<65 years	7.36 (4.76–9.97)	0.65 (0.19–2.18)	0.493	13.33 (6.80–19.86)	0.96 (0.28–3.22)	0.950
	≥65 years	5.30 (1.86–8.73)			9.30 (0.71–17.88)		
ECOG-PS	0–1	7.36 (4.35–10.37)	0.61 (0.28–1.32)	0.215	13.93 (0.26–27.60)	0.58 (0.26–1.30)	0.191
	2	4.6 (0.00–10.76)			9.56 (1.02–18.11)		
Metastatic	De novo	4.96 (0.93–8.99)	1.23 (0.54–2.81)	0.616	8.86 (0.00–19.84)	1.35 (0.57–3.17)	0.486
disease	Recurrence	7.60 (3.37–11.82)			13.3 (6.84–19.82)		
Soft tissue	Absent	5.83 (2.60–9.06)	1.63 (0.77–3.47)	0.200	8.86 (7.16–10.57)	1.57 (0.72–3.44)	0.254
metastasis	Present	7.6 (0.00–15.82)			14.83 (7.77–21.89)		
Lung metastasis	Absent	7.60 (3.88–11.31)	0.48 (0.22–1.06)	0.073	14.83 (12.07–17.59)	0.55 (0.25–1.23)	0.152
	Present	5.83 (1.62–10.04)			8.30 (4.34–12.25)		
Liver metastasis	Absent	7.60 (6.19–9.00)	0.60 (0.26–1.40)	0.244	13.93 (5.53–22.32)	0.62 (0.26–1.46)	0.280
	Present	4.96 (2.82–7.10)			9.56 (7.52–11.61)		
Bone metastasis	Absent	5.53 (2.55–8.51)	1.26 (0.53–2.98)	0.592	13.93 (6.84–21.02)	0.79 (0.33–1.91)	0.608
	Present	8.03 (6.74–9.32)			8.86 (7.11–10.62)		
Peritoneum	Absent	7.13 (4.56–9.69)	1.27 (0.48–3.40)	0.625	9.93 (3.40–16.46)	1.27 (0.43–3.73)	0.655
metastasis	Present	4.60 (0.00–18.84)			17.56 (0.00–42.65)		
Surgical	Yes	8.30 (0.00–19.16)	0.74 (0.34–1.60)	0.446	13.33 (5.01–21.65)	0.74 (0.33–1.66)	0.473
treatment	No	5.30 (1.51–9.08)			9.56 (2.80–16.33)		
Radiotherapy	Yes	8.30 (2.91–13.68)	0.74 (0.33–1.64)	0.460	13.33 (6.95–19.71)	0.63 (0.27–1.45)	0.284
	No	4.96 (2.16–7.77)			8.86 (0.00–18.99)		
HIF-1 alpha	<100	4.96 (3.43–6.50)	2.78 (1.20–6.30)	**0.014**	8.30 (5.08–11.51)	3.44 (1.40–8.30)	**0.006**
score	≥100	12.90 (7.59–18.20)			20.36 (15.98–24.74)		

*Univariate cox regression analysis. Bold values indicate statistical significance at *P* < 0.05 level. Data are presented as median (CI). CI: Confidence interval; ECOG-PS: Eastern Cooperative Oncology Group-performance status; HR: Hazard ratio; mOS: Median overall survival; mPFS: Median progression-free survival; HIF-1: Hypoxia-inducible factor-1.

**Figure 4.**
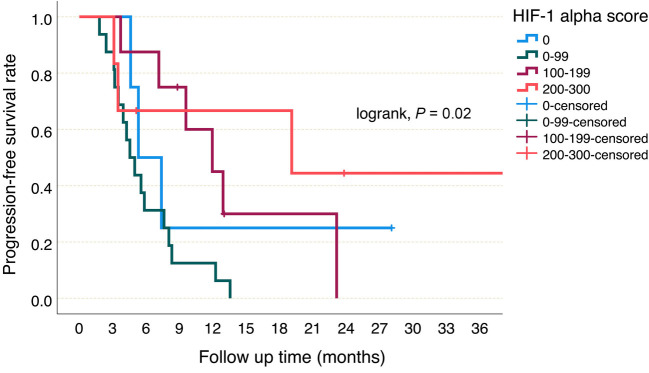
**Progression-free survival according to HIF-1 alpha scores.** HIF-1: Hypoxia-inducible factor-1.

**Figure 5.**
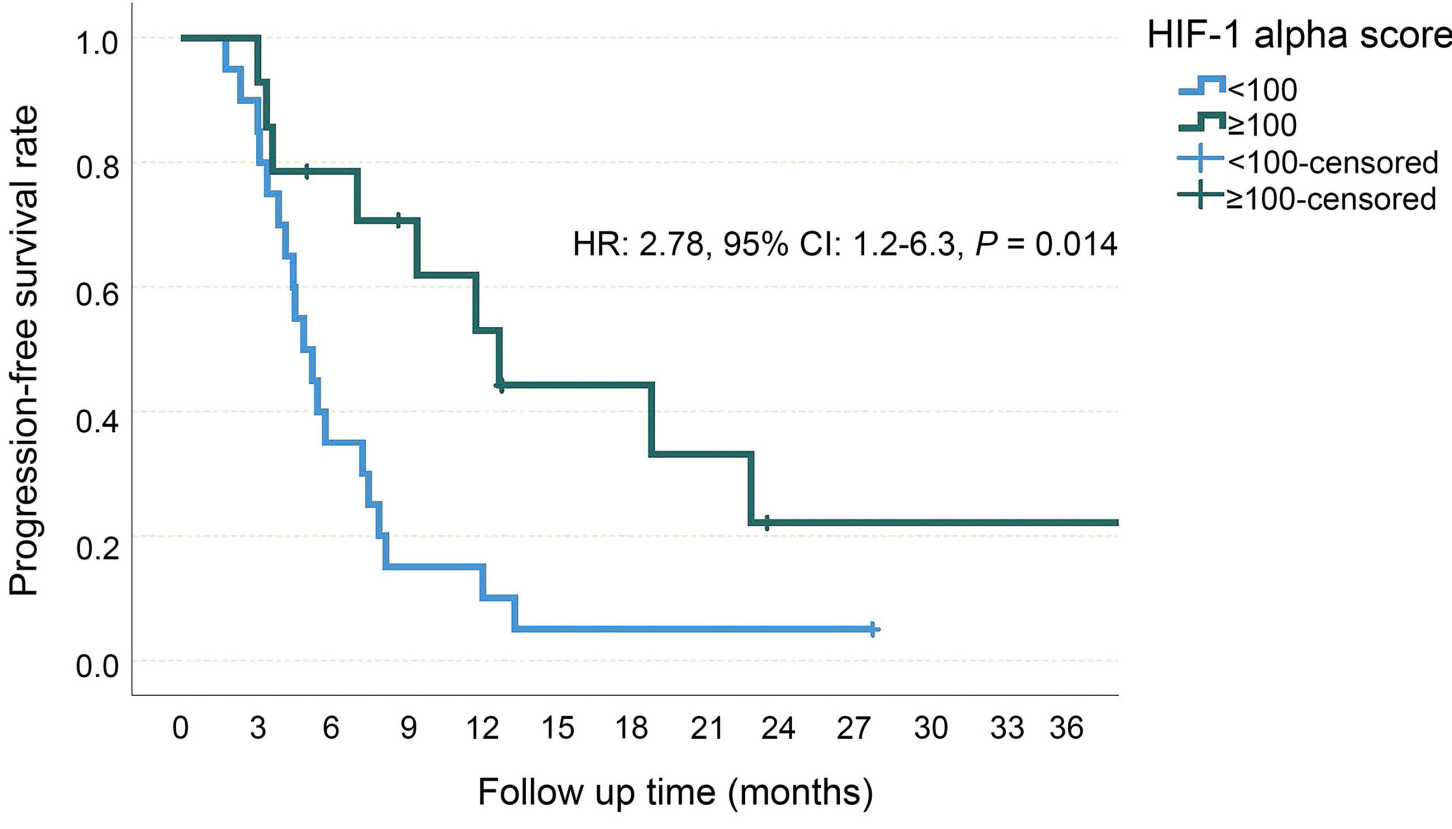
**Progression-free survival of low and high HIF-1 alpha score groups.** HIF-1: Hypoxia-inducible factor-1; HR: Hazard ratio; CI: Confidence interval.

**Figure 6.**
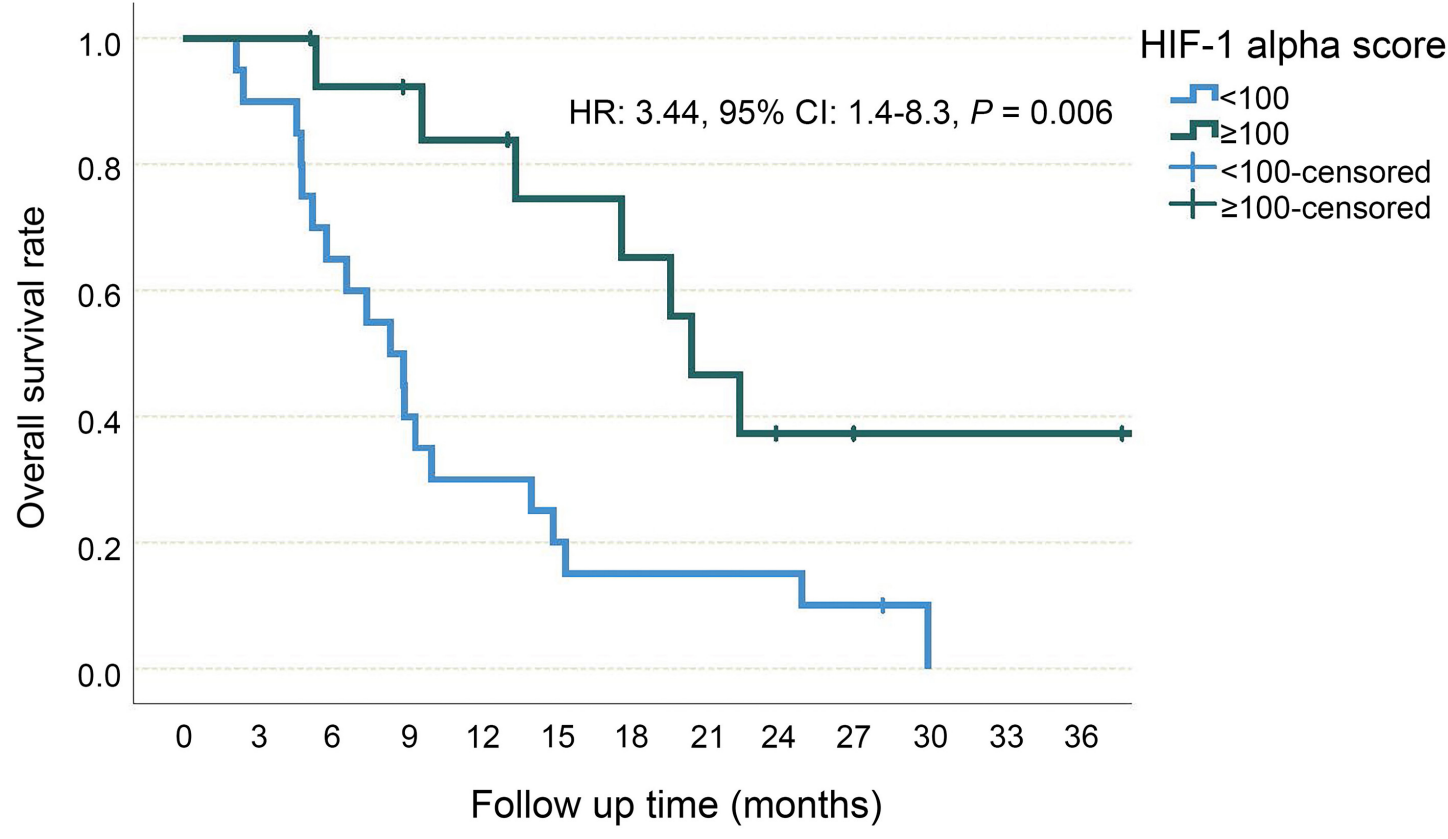
**Overall survival of low and high HIF-1 alpha score groups.** HIF-1: Hypoxia-inducible factor-1; HR: Hazard ratio; CI: Confidence interval.

The median PFS was 5.3 (95% CI 2.6–8.0) months in patients with a HIF-1alpha score of 0, 4.5 (95% CI 3.1–6) months in patients with a HIF-1 alpha score between 1 and 99, 11.9 (95% CI 6.1–17.7) months in patients with a HIF-1 alpha score between 100 and 199 and 19.1 (95% CI 0–49.6) months in patients with HIF-1 alpha score 200 or above (*P* ═ 0.02, Figure 4). Patients with a HIF-1 alpha score below 100 were considered as HIF-1 alpha low score and patients with a HIF-1 alpha score of 100 and above were considered as HIF-1 alpha high score. The median PFS was found to be 4.9 (95% CI 3.4–6.5) months in the HIF-1 alpha low group, while it was 12.9 (95% CI: 7.6–18.2) months in the HIF-1 alpha high group (HR 2.78, 95% CI 1.2–6.3, *P* ═ 0.014, Figure 5). On the other hand, the median OS was 7.4 (95% CI 2.9–11.8) months in patients with a HIF-1 alpha score of 0, 8.3 (95% CI 3.8–12.8) months in patients with a HIF-1 alpha score between 1 and 99, 20.4 (95% CI 8.7–32) months in patients with a HIF-1 alpha score between 100 and 199, and 22.3 (95% CI 16.4–28.2) months in patients with HIF-1 alpha score 200 or above (*P* ═ 0.025). In the HIF-1 alpha low group, median OS was 8.3 (95% CI 5.1–11.5) months, while in the HIF-1 alpha high group, it was 20.4 (95% CI 15.9–24.7) months, (HR 3.44, 95% CI 1.4–8.3, *P* ═ 0.006, Figure 6).

## Discussion

To the best of our knowledge, this is the first study on the prognostic importance of HIF-1 alpha expression in patients diagnosed with metastatic cervical SCC. Our findings indicate that low expression of HIF-1 alpha correlates with a poorer prognosis in patients treated with bevacizumab in combination with standard chemotherapy as their initial therapy.

Mammalian cells need oxygen homeostasis to maintain aerobic metabolism and energy production [18]. Hypoxia occurs frequently in many solid tumors due to rapid proliferation of the tumor and obstruction or compression of blood vessels. Tumor cells in the hypoxic region activate different pathways for adaptation when faced with low oxygen levels. HIF-1 transcription factor activation is the best-known adaptation mechanism in the low-oxygen microenvironment [19]. Activated HIF-1 alpha is effective in glucose metabolism, cell proliferation, migration, and angiogenesis [19]. There are studies showing that HIF-1 alpha expression in solid tumors is associated with poor prognosis [15, 20, 21]. In our investigation, we discovered a positive correlation between PFS and OS and the HIF-1 alpha level. This result may have occurred because our patients received bevacizumab, an antibody developed against the VEGF molecule that acts on the HIF-1 alpha pathway.

Studies have been conducted on the relationship between HIF-1 alpha expression and cervical cancer prognosis. In a study that included cervical intraepithelial neoplasia, carcinoma in situ, cervical cancer and matched nonadjacent normal tissues, it was found that high HIF-1 alpha expression was more common in cancer patients and the 5-year survival time was poor in patients expressing HIF-1 alpha [22]. However, only five stage-IV patients were included in this study. In another study, only patients diagnosed with early-stage cervical cancer were included, and disease-free survival was found to be significantly worse in patients with high HIF-1 alpha expression [23]. In these two studies, patients diagnosed with early-stage cervical cancer were included and the anti-VEGF antibody bevacizumab, which is found in the HIF pathway and increases angiogenesis, was not used. This situation is similar to the prognostic role of HER2 expression in breast cancer. While historically, HER2 expression has been an unfavorable prognostic factor in breast cancer, significant improvements in PFS and OS have been observed with the use of anti-HER2-based therapies, and in cervical cancer, treatments targeting VEGF, a mediator of the HIF1 pathway, may have eliminated the negative role of HIF1 expression [24]. VEGF pathway has become a target in advanced-stage cervical cancer. In the GOG 240 trial, the addition of bevacizumab to standard platinum-based chemotherapy showed a contribution to PFS and OS. PFS was 8.2 months in the bevacizumab-added arm, while it was 5.9 months in the standard chemotherapy arm [25]. In our study, the PFS in the group with low HIF-1 alpha scores was around five months and despite the addition of bevacizumab, it was similar to the historical data of standard chemotherapy. In the Keynote 826 trial investigated the contribution of pembrolizumab to standard chemotherapy, nearly 40% of the patients did not receive bevacizumab treatment [14]. Having received bevacizumab treatment did not make a difference in terms of the effectiveness of pembrolizumab added to standard platinum-based chemotherapy (both PFS and OS). Considering that bevacizumab increases the frequency of serious side effects, studies are needed to predict in which patients this treatment will be more effective. In our study, we included only patients who received bevacizumab, because the combination of bevacizumab and chemotherapy is one of the standard treatment options in current guidelines, and patients who did not receive bevacizumab treatment may have reasons that would affect the results, such as fistula, resistant hypertension, or a history of thromboembolic events. Since we did not include patients who did not receive bevacizumab, we cannot evaluate the predictive role of HIF-1 alpha expression, but in our study, the PFS and OS obtained in the group with low HIF-1 alpha scores were similar to historical data obtained with chemotherapy, suggesting that these patients did not benefit from bevacizumab.

The study’s main limitations include its retrospective nature and the small number of patients involved. Additionally, the absence of bevacizumab-naive patients in the study groups prevents the assessment of the predictive role of HIF-1 alpha expression.

## Conclusion

Our research is the inaugural study to elucidate the correlation between HIF-1 alpha expression and the efficacy of first-line treatment in metastatic cervical SCC. With the emerging role of immune checkpoint inhibitors in cervical cancer treatment, there arises a critical need for predictive studies to determine the patients for whom bevacizumab administration would be most beneficial.
